# Child and adolescent mental health problems in Nepal: a scoping review

**DOI:** 10.1186/s13033-019-0310-y

**Published:** 2019-08-12

**Authors:** Ashmita Chaulagain, Arun Kunwar, Sarah Watts, Anthony P. S. Guerrero, Norbert Skokauskas

**Affiliations:** 1 Regional Center for Child and Adolescent Mental Health and Child Protection, Norwegian University of Science and Technology, Trondheim, Norway; 2 Child and Adolescent Psychiatry, Kanti Children Hospital, Kathmandu, Nepal; 3 Department of Mental Health and Substance Abuse, World Health Organization, Geneva, Switzerland; 4 Child and Adolescent Psychiatry Division, University of Hawai’i John A. Burns School of Medicine, Honolulu, USA

**Keywords:** Child and adolescent, Psychiatry, Mental health, Mental disorder, Nepal

## Abstract

**Introduction:**

Globally, 10–20% of children and adolescents suffer from mental disorders, with half of all them starting by the age of 14 and three-quarters before the age of 25. In Nepal, 40% of the population is younger than 18 years of age, and as such there is a large proportion of the population that is at risk of developing a mental disorder. There has been a recent recognition of child and adolescent mental health problems in Nepal, although prior to this it had remained almost invisible on the health agenda. In response to growing concern, there is a need to conduct a review on children and adolescent mental health problems in Nepal.

**Objective:**

To review the existing studies on child and adolescent mental health problems in Nepal.

**Methodology:**

A scoping review approach was used to identify studies on child and adolescent mental problems in Nepal. A search of Medline and PubMed databases for articles published from the database inception to August 2018 was conducted.

**Results:**

Ten papers were identified, and they all together included 7876 participants. Two studies reported on Post traumatic Stress Symptoms (PTSS) and described a prevalence of 10.7% to 51% of earthquake-affected children and adolescents in the Kathmandu district of Nepal. Another study reported that 53.2% of former child soldiers met the cut-off score for PTSS. Two school surveys found that the prevalence of emotional and behavioural problems in school children ranged between 12.9 and 17.03%, whereas a study on emotional and behavioural disorders in homeless children reported a prevalence of 28.6%. The prevalence of Autism Spectrum Disorder (ASD) was estimated to be as high as three in every 1000 persons in Nepal by one study. The clinical prevalence of anxiety disorders was reported ranging from 18.8% to 24.4% while that of Attention Deficit Hyperactivity Disorder (ADHD) was 10–11.7% in various clinical samples of children and adolescents.

**Conclusion:**

Only a few studies on the prevalence of child and adolescent mental health in Nepal have been conducted. Clearly, there is a need for better study design and larger studies to understand more fully the prevalence of child and adolescent mental health disorders in Nepal, in order to adequately plan public health services accordingly.

**Electronic supplementary material:**

The online version of this article (10.1186/s13033-019-0310-y) contains supplementary material, which is available to authorized users.

## Introduction

Child and adolescent mental disorders are common and linked to pre-mature death and serious dysfunction in adult life [1]. About half of all mental disorders start by the age of 14 years and three-quarters before the age of 25 [1, 2]. Worldwide prevalence rates of child and adolescent mental disorders are around 10–20%, with similar types of disorders, such as anxiety disorder, behaviour disorder and mood disorders seen across cultures [2]. Nepal is a low-income country with a total population of approximately 20 million, of whom 40% (12 million) are younger than 18 years of age [3, 4]. The Ministry of Health and Population of Nepal estimates that about 15–20% of this population (2–3 million) may suffer from some form of mental disorder [4, 5].

Mental health is shaped to a great extent by social, economic and environmental factors [6]. Exposure to a range of environmental adversities increases the risk of disorders in children through the biological embedding of environmental risk. Poverty, a lower social position in society, war and exposure to violence in neighbourhoods have all been shown to have negative influences on the development of child psychopathology [7]. Unfortunately, many more children and adolescents in Nepal are exposed to such factors, and often more so than their peers in high income countries. For example, almost half (41.6%) of all children in Nepal are living under multidimensional poverty as measured across health, education and living standards [8]. Children from poorer backgrounds are likely to have a greater exposure to child labour, exploitation and human trafficking, domestic violence and sexual abuse [4, 9–12]. Moreover, the changing family structure due to divorce, separation from joint family to nuclear family, parental neglect and parental substance abuse also put children at a higher risk of psychosocial and mental health problems [12].

Natural disasters like earthquakes, floods, and landslides are common in Nepal [13, 14]. The massive earthquake of 2015 directly affected 1.7 million children in Nepal [13]. Such disasters lead to displacements, disappearances, injuries and death affecting families, children and their mental well-being. Despite this, child and adolescent mental health problems and disorders have been unacknowledged for many years in Nepal.

Only recently, has there been greater importance given to identifying and treating mental disorders in children [4, 5]; however, the magnitude of child and adolescent mental problems in Nepal is still not clear. There are many reasons for this, including the absence of a child and adolescent mental health policy, poor child and adolescent mental health services (there is only one outpatient child and adolescent mental health clinic in the whole country and no inpatient facilities), as well as an acute shortage of child and adolescent psychiatrists and allied professionals (only one child and adolescent psychiatrist in the whole country). Furthermore, no specialized postgraduate training in child and adolescent psychiatry is available in Nepal and limited research is performed [4, 15–18]. The Government of Nepal has allocated less than 1% of its total health budget for mental health; child and adolescent mental health services receive a negligible portion of this amount [18, 19].

This paper aimed to provide an overview of child and adolescent mental health problems in Nepal.

## Methodology

This study used a scoping review approach and employed the following inclusion criteria: any type of study reporting on mental health disorders in children and adolescents, conducted in Nepal, published in English or Nepali. Two databases (PubMed and Medline were searched from their inception to August 2018, using terms ‘mental disorder’, ‘child and adolescent’ and ‘Nepal’). Titles and abstracts were examined using the inclusion criteria, after which full articles were retrieved.

According to Preferred Reporting Items for Systematic Review and Meta-Analyses Extension for Scoping Review Guideline (PRISMA-ScR), the critical appraisal of the included studies is an optional item [20]. However, we performed the critical appraisal taking into account that it will help to consider the methodological quality of the included studies while interpreting the findings from those studies. For critical appraisal, the methodological quality assessment tool of the National Heart, Lung, and Blood Institute (NHLBI) Quality Assessment Tool for Cross-Sectional Studies, [21] and the Guidelines for evaluating prevalence studies [22] were used. Given that, this was a comprehensive scoping review, and not a systematic review, one reviewer performed the quality assessment, and another co-author later confirmed it. There were no articles excluded based on the quality criteria.

## Results

### Study selection

**Fig. 1:**
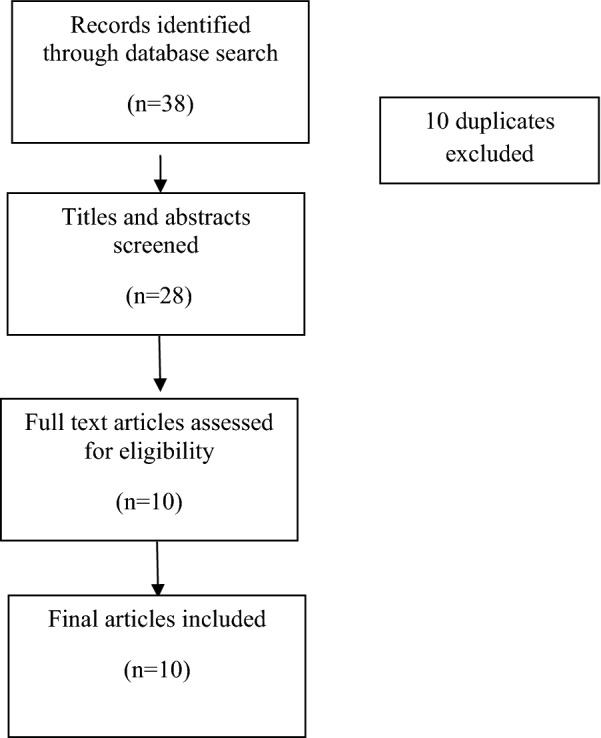
Schematic representation of the literature search

The initial online search produced 38 articles. Duplicates were removed and this reduced the number of articles to 28. The titles and abstracts of these 28 studies were then reviewed, and 18 were excluded because they were either not related to mental health disorders, and/or they were not related to mental health disorders in children and adolescents, and/or were not conducted in Nepal. As such, 10 studies were identified that met the inclusion criteria (see Fig. 1).

### General description

Most of the studies (n = 7/10) were population studies conducted in the community setting [23–26] and schools [27–29]. Most of the studies were cross-sectional in nature [23, 25–27] and one was a cross-sectional cohort study [24]. The sample size of these studies ranged ranged from 126 to 4098 (Tables 1 and 2).

**Table 1:** Epidemiological study of child and adolescent mental health problems in Nepal

Study	Study design	Diagnostic tool	Study sample	Sample size (n)	Age range	Prevalence
Acharya et al. [23]	Cross-sectional study	Trauma exposure questionnaire and Child PTSD scale (CPSS)	Community-base sample	800	7–16	PTSD-51% among school-aged children in Kathmandu district
Kohrt et al. [24]	Cohort study	Depression Self Rating Scale SCARED-5 Child PTSD symptom scale (CPSS) Strength and Difficulties Questionnaire Function Impairment tool	Former child solider vs Community-base sample	Case 141 former child soldiers, 141 never conscripted children	Not available	53.2% of child soldiers had depression, anxiety-46.1%, PTSD symptoms-55.3%, Psychological difficulties-39%, and function impairment-62.4% Child soldiers had greater odds of meeting cutoff for depression (OR = 3.56, 95% CI 2.33–5.43), PTSD, Psychological difficulties (OR = 2.91, 95% CI 1.53–5.51), and function impairment (OR = 2.04, 95% CI 1.41–2.96)
Ojha et al. [25]	Cross-sectional study	Child Behaviour Checklist 6.18	Homeless children	126	6–18	Emotional and behavioural problem-28.6% Conduct problem was most common among male children 8.77%, followed by ODD 5.26%, ADHD 3.5%, Anxiety 3.50% Anxiety was most common in female children 13.04%, followed by depression 7.24%, conduct 4.34%
Heys et al. [26]	Cross sectional	Autism Quotient-10 translated version in Nepali	General Population	4098	10–13	14 out of 4098 children scored greater than 6 out of 10, indicative of autistic symptomatology Estimated true prevalence of Autism is 3 in per 1000 (95% CI 2–5 in 1000)
Silwal et al. [27]	Cross-sectional study	Child Posttraumatic-Stress Disorder Scale (CPSS) Depression Self Rating Scale (DSRS)	School-based sample	893	11–17	The prevalence of PTSS in the Sindhupalchok and Kathmandu districts were 39.5% and 10.7%, and depression symptoms were 40.4% and 23.2% respectively
Timalsina et al. [28]	Cross-sectional study	Paediatric Symptoms Checklist (Y-PSC)	School-based sample	287	12–19	12.09% of the adolescent had psychosocial problems While categorizing the psychosocial problems, Internalizing problems (44.6%), ADHD (25.8%) and externalizing problems (4.2%)
Bista et al. [29]	Cross-sectional study	Paediatric Symptoms Checklist (Y-PSC)	School-based sample	787	11–19	17.03% of adolescent had psychosocial dysfunction

**Table 2:** Clinical study of child and adolescent mental disorder in Nepal

Study	Study design	Diagnostic tool	Sample size	Age range	Prevalence
Rimal et al. [30]	Cross-sectional study	Strength and Difficulty Questionnaire Spence Anxiety Scale Vanderbilt rating scale for ADHD	85	Not available	Speech and language delay-22.4% Behavioral problem- 21.2%, anxiety disorders-18.8%, cerebral palsy-14.1% global developmental delay-11.8%, ASD-10.6% ADHD-10%, Adjustment problem-8.2%, Depression-4.7%, Conversion disorder-9.4%
Rimal and Pokahrel [31]	Cross-sectional study	ADHD rating scale with DSM-IV classification Spence anxiety scale child and parent rated version Strength and Difficulty Questionnaire	350	Not available	The yearly prevalence of ADHD in clinical sample was-11.7% with male: female ratio of 4:1 Associated comorbidities: Sleep problem 12 (29.3%), Learning difficulty 10 (24.4%), Anxiety disorder 10 (24.4), Oppositional Defiant Disorder 9 (22%), Autism Spectrum Disorder 5 (12%), speech delay 6 (14.6%), and 4 (10%) had associated tics
Risal et al. [32]	Retrospective study	ICD-10	168	Below 18 years of age	Dissociative disorder (15%), Seizure disorder (15%), Depressive disorder (13.8%), Intentional Self Harm (13.8%)

### Critical appraisal within sources of included studies

Only two studies [23, 29] were of good quality, six remaining being scored as “poor quality” [25, 26, 28, 30–32] and two were of fair quality [24, 27] (Additional file 1: Table S1).

In terms of sampling method, only two studies used probability-sampling technique [23, 29] while remaining eight used convenient sampling technique [24–28, 30–32]. Only one study provided sample size justification, power description, or variance and effect estimates [29]. Only four studies used validated criteria for data collection [23, 24, 27, 28] The quality scores of the included studies are shown in Additional file 1: Table S1.

More information will now be described about each of the ten studies, including the screening tool used, the prevalence rates reported, and the context of the study.

### The Gorkha Earthquake-Post Traumatic Stress Symptoms (PTSS)

Following the massive earthquake, also known as the Gorkha earthquake, in 2015 that directly affected 1.7 million children and adolescents, two studies assessed post-traumatic stress symptoms (PTSS) among children and adolescents using the child PTSD symptom scale (CPSS) [23, 27]. The CPSS is a psychological screening tool that comprises a set of signs and facilitates the recognition of PTSS. One study was conducted in the Kathmandu district in a community setting [23] while another study was conducted in schools in the Kathmandu and Sindupalchowk districts [27]. The rate of PTSS in Kathmandu district reported in these two studies was 10.7% [27] and 51% [23], respectively. The rate of PTSS in Sindupalchowk district was 39.5% [27].

### Former child soldiers-PTSD symptoms, depression and anxiety

One cohort study assessed mental health problems, i.e. symptoms of PTSD, depression symptoms and anxiety symptoms among former child soldiers in comparison with children never conscripted to an armed group. The study found that more child soldiers were above the cut-off scores for each mental health scale compared with never conscripted children. PTSD symptoms were assessed using CPSS; it was found that the percentage of former child soldiers meeting cut-off scores for PTSD symptoms was 55.3% (n = 78) vs. 20% (n = 28) among never-conscripted children. Similarly, depression symptoms were assessed using the Depression Self Rating Scale (DSRS); 53.2% (n = 75) of former child soldiers vs. 24.1% (n = 34) of never-conscripted children met the cut-off score for depression symptoms. Anxiety symptoms were assessed using the Screen for Child Anxiety Related Emotional Disorders (SCARED-5); the proportion of children meeting the cut-off score was 46.1% (n = 65) in former child soldiers vs. 37.6% (53) in never-conscripted children [24].

### Children and homelessness—emotional and behavioural disorders

One of the studies assessed emotional and behavioural problems among homeless children and adolescents (n = 126). This study conducted the assessment in two stages. At the first stage, emotional and behavioural problems were assessed using the Child Behaviour Checklist (CBCL), while in the second stage, a diagnosis was made by psychiatrist using DSM-IV-TR criteria The prevalence of emotional and behavioural problems among homeless children was 28.57% (26.31% in boys and 30.43% in girls) based on the CBCL and 23.01% (21.05% in boys and 24.63% in girls) based on the final diagnosis. Anxiety disorders were more common in girls (13.04%) than boys (3.5%), while conduct disorders were more common among boys (8.77%) than girls (4.34%). Depression was present only among girls (7.24% vs. 0%) while ADHD (3.5% vs. 0%) and Oppositional Defiant Disorder (5.26% vs. 0%) were present only among boys as assessed by DSM-IV-TR criteria [25].

### Community and school-based studies—autism, emotional and behavioural problems and ADHD

Heys et al. translated, adapted and tested the acceptability of a Nepali language version of a screening tool for autism (Autism Quotient-10). Using this tool, they estimated the prevalence of autism in rural Nepali children aged 9–13 years. Fourteen out of 4098 children scored > 6 out of 10, indicative of elevated autistic symptomatology, of which 13 also screened positive for disability. This study estimated the prevalence of autism as being three per 1000 children and adolescents [26].

Two of the ten reviewed studies assessed emotional and behavioural problems among school attending adolescents using the Paediatric Symptoms Checklist [28, 29]. Emotional and behavioural problems ranged between 12.09% [28] and 17.03% [29]. The rate of internalising problems in one of the school-based studies was 44.6%, while that of externalising problems was 30%. Attention deficit hyperactivity problem was present among 25.8% of school-attending adolescents [29].

### Clinical studies-autism, emotional and behavioural problems and ADHD

Rimal et al. [30] assessed developmental and behavioural problems among children in a clinical setting. Standard screening and assessment tools, i.e. the Strength and Difficulty Questionnaire (SDQ), Spence anxiety scale and Vanderbilt rating scale for attention deficit hyperactivity problem were used. The most common problems were speech and language delay (22.4%), behavioural problems (21.2%), anxiety problem (18.8%) and attention deficit hyperactivity problem (10%) [30].

Rimal et al. [31] also assessed the rate of Attention Deficit Hyperactivity Disorder (ADHD) via ADHD rating scale with diagnostic criteria consistent with DSM-IV classification and associated co-morbidities among school children. The study found an ADHD prevalence of 11.7%. The most common co-morbidities were sleep problems (29.3%), learning difficulties and anxiety disorders (24.4% each), oppositional defiant disorder (22%), speech delay (14.6%) and autism spectrum disorder (12%) [31].

Risal et al. [32] conducted a retrospective study to identify psychiatric disorders in patients below 18 years of age who presented to a psychiatric out-patient unit. The diagnosis made by the psychiatric consultant using ICD criteria reported that dissociative disorders (15%) and seizure disorders (15%) were the most common diagnoses, followed by depressive disorder and intentional self-harm (13.8% each) [32].

## Discussion

This paper has provided an overview of child and adolescent mental health problems in Nepal. Only ten eligible studies were identified. Among these, seven were population studies, while three studies assessed clinical samples.

There is a small but growing evidence base on child and adolescent mental health problems and disorders in Nepal [23–32]. Unfortunately, all studies were relatively small. The most common mental health problems assessed among children and adolescents of Nepal as found in this review are PTSS symptoms, followed by emotional and behavioural problems. Likewise, children and adolescents exposed to the aftermath of the recent earthquake, those involved in war, as well as homeless and school-going children are the most well-studied groups so far.

The rate of PTSS symptoms among earthquake-affected children and adolescents of the Kathmandu district reported in this review varied greatly (10.7% to 51%). These wide variations could be due to differences in methodology. The study that reported a higher rate of PTSS was conducted among both children and adolescents [23], while the study that reported a lower prevalence assessed only adolescents [27]. Likewise, there was difference in the study settings in these two studies, as one of the studies was a community-based study that adopted multistage cluster sampling and including 800 earthquake-affected children and adolescents [23], while the other was a school-based study conducted in three schools and including 440 adolescents [27]. However, the prevalence rate of PTSS symptoms (51%) is similar to the prevalence rate of 47.7% among children 3 months after a major earthquake in Turkey [33] and to the prevalence rate of 44% among children 1 year after a major earthquake in China [34].

The prevalence of emotional and behavioural problems among sheltered homeless children was 28.57% [25], which is in the range of the rates of 24% to 40% in a meta-analysis on mental illness in homeless children [35]. Consistent with previous studies, this study showed that internalising problems are more common in girls than boys, and the opposite for externalising problems [35, 36].

Heys et al. estimated that the prevalence of autism was 0.3% [26], which is lower compared to the estimated prevalence of 1.89% in South Korea [37]. This difference might be due to differences in methodology. The study in Nepal used the AQ-10 screening tool in 4098 children and adolescents while the study in South Korea used the Autism Spectrum Screening Questionnaire in 55,266 children and adolescents.

The prevalence of psychosocial problems among adolescents in a school-based sample ranged from 12.09 to 17.03% [28, 29]. This prevalence is similar to the rate of 14.3% found in Sub-Saharan Africa school age children, as shown by one the meta-analysis [38].

The prevalence of ADHD in one of the hospital-based studies in Nepal was 10% [30], which is similar to the rate of 11% among children in a hospital-based study in Uganda [39]. However, this is less than the prevalence of 20.3% among children in a hospital-based study in India [40].

The studies included in this review were not free from limitations. Only two out of ten studies were rated as of good quality. Most of the included studies were cross-sectional and descriptive in nature, conducted with small samples collected through convenient sampling techniques and different settings; therefore, the findings of the study cannot be generalised to the entire population of children and adolescents of Nepal. In addition, most of the screening tools used for these studies were not validated for use in a Nepalese context, which might be also be considered as a threat to both the internal and external validity of the study findings. Very few population studies have studied mental health problems and disorder among children and adolescents in Nepal, so this scoping review included the available clinical studies conducted to assess common mental disorders among children and adolescents in clinical samples. Hence, the findings from clinical studies included in this scoping review should be interpreted cautiously as they do not provide an estimate of actual disease prevalence in the general population.

### Strength and limitation of the study

According to our knowledge, this is the first scoping review that has provided an overview child and adolescent mental health problems in Nepal. This study has also provided the quality assessment of the included studies. However, the search was limited to only two databases for convenience and therefore some relevant studies might not have been identified. However, given the dearth amount of literature on this important topic, these findings remain useful.

### Implications of the study

The findings of this study have implications for policy initiatives and service delivery. Given the context of Nepal where there is a lack of child and adolescent mental health plan and policy, the findings of the prevalence of child and adolescent mental health problem in this study suggest that there is a need of child and adolescent mental health policy and plan in the country. Policy makers and service delivery should explore and implement evidence-based approach for promoting and protecting child and adolescent mental health. They should create a competitive mental health workforce that can address the existing child and adolescent mental health problems.

### Conclusion

1. The existing literature demonstrates that mental health problems and disorder are common among the children and adolescents of Nepal. However, methodological variations, poor quality and constraints across those studies make it difficult to reach firm conclusions on the true prevalence of mental health disorders in children and adolescents in Nepal and suggest a huge research gap in the field of child and adolescent mental health in the country.
2. There have been no national level prevalence studies on child and adolescent mental health problems and disorders in Nepal.
3. More research that is robust is required to assess the prevalence of child and adolescent mental health problems and disorder.
4. Future research should use total population or representative sample and valid screening and diagnostic tool and contribute toward providing the true prevalence of different child and adolescents mental health problems and disorder in the country.

## Additional file

**Additional file 1: Table S1.** Quality assessment of included studies.

## Data Availability

Not applicable

## Abbreviations

ADHD: Attention Deficit Hyperactivity Disorder
CBCL: Child Behaviour Checklist
DSM-IV: Diagnostic Statistic Manual-IV
DSRS: Depression Self Rating Scale
PRISMA-ScR: Preferred Reporting Items for Systematic Review and Meta-Analyses Extension for Scoping Review Guideline
PTSS: post-traumatic stress symptoms
SDQ: Strength and Difficulty Questionnaire
