# Supplementary material for: Child and adolescent mental health problems in Nepal: a scoping review
Source: Int J Ment Health Syst. 2019 Aug 12;13:53. doi: 10.1186/s13033-019-0310-y (PMC6689861; doi:10.1186/s13033-019-0310-y)
Supplement: Supplementary file 1 — Additional file 1: Table S1. Quality assessment of included studies. [file 13033_2019_310_MOESM1_ESM.docx]

**Table S1: Quality assessment of included studies**

|  | Assessment items | | | | | | | | | Quality  rating |
| --- | --- | --- | --- | --- | --- | --- | --- | --- | --- | --- |
| Study | Clearly stated research question or objective | Clearly defined study population | Ascertainment method used (probability sampling or entire population surveyed | Subjects selected or recruited from the same or similar populations/ Inclusion and exclusion criteria for being in the study pre specified and applied uniformly to all participants | Sample size justification, power description, or variance and effect estimates provided | Sufficient timeframe to see an association between exposure and outcome | Outcome measures clearly defined, valid, reliable, and implemented consistently across all study participants | Response rate >70% | Adjustment for confounders |  |
| Acharya etal, (2017) | Yes | Yes | Yes | Yes | No | Yes | Yes | Yes | Yes | Good |
| Kohrt et.al.(2008) | Yes | Yes | No | Yes | No | Yes | Yes | Yes | Yes | Fair |
| Ojha et.al (2013) | Yes | Yes | No | Yes | No | NR | No | Yes | No | Poor |
| Heys et.al. (2018) | Yes | No | No | Yes | No | NR | CD | No | NR | Poor |
| Silwal et al. (2018) | Yes | Yes | No | Yes | No | Yes | Yes | Yes | No | Fair |
| Timalsina et.al. (2018) | Yes | No | No | Yes | No | NR | Yes | Yes | No | Poor |
| Bista et.al.(2016) | Yes | Yes | Yes | Yes | Yes | NR | No | Yes | Yes | Good |
| Rimal et al. (2014) | No | No | No | Yes | No | NR | No | Yes | NR | Poor |
| Rimal et al. (2016) | Yes | No | No | Yes | No | NR | No | Yes | No | Poor |
| Risal et.al. (2010) | No | Yes | No | Yes | No | NR | No | Yes | NR | Poor\| |
